# Early Care and Education Center Environmental Factors Associated with Product- and Process-Based Locomotor Outcomes in Preschool-Age Children

**DOI:** 10.3390/ijerph19042208

**Published:** 2022-02-15

**Authors:** Jacob Szeszulski, Elizabeth Lorenzo, Michael Todd, Teresia M. O’Connor, Jennie Hill, Gabriel Q. Shaibi, Sonia Vega-López, Matthew P. Buman, Steven P. Hooker, Rebecca E. Lee

**Affiliations:** 1 Institute for Advancing Health through Agriculture, Texas A&M AgriLife Research, 17360 Coit Rd., Dallas, TX 75252, USA; 2 Center for Health Promotion and Disease Prevention, Edson College of Nursing and Health Innovation, Arizona State University, 550 North 3rd St., Phoenix, AZ 85004, USA; ellorenz@utmb.edu (E.L.); gabriel.shaibi@asu.edu (G.Q.S.); 3 College of Health Solutions, Arizona State University, 550 North 5th St., Phoenix, AZ 85004, USA; sonia.vega.lopez@asu.edu (S.V.-L.); mbuman@asu.edu (M.P.B.); relee6@asu.edu (R.E.L.); 4 School of Nursing, University of Texas Medical Branch, 1114 Mechanic St., Galveston, TX 77555, USA; 5 College of Nursing and Health Innovation, Arizona State University, 550 North 3rd St., Phoenix, AZ 85004, USA; mike.todd@asu.edu; 6 USDA/ARS Children’s Nutrition Research Center, Department of Pediatrics, Baylor College of Medicine, 1100 Bates Ave., Houston, TX 77030, USA; teresiao@bcm.edu; 7 Department of Population Health Sciences, University of Utah, 201 Presidents’ Cir, Salt Lake City, UT 84112, USA; jennie.hill@hsc.utah.edu; 8 Southwestern Interdisciplinary Research Center, Arizona State University, 400 E. Van Buren St., Suite 800 Phoenix, Phoenix, AZ 85004, USA; 9 College of Health and Human Services, San Diego State University, 5500 Campanile Dr., San Diego, CA 92182, USA; shooker@sdsu.edu

**Keywords:** Latinos, social ecology, child day care centers

## Abstract

Environmental characteristics of early care and education centers (ECECs) are an important context for preschool-aged children’s development, but few studies have examined their relationship with children’s locomotor skills. We examined the association between characteristics of the ECEC environment with quantitatively (i.e., product-based) and qualitatively (i.e., process-based) measured locomotor skills, using the Progressive Aerobic Cardiovascular Endurance Run (PACER) and the locomotor portion of the Children’s Activity and Movement in Preschool Study (CHAMPS) motor skills protocol (CMSP), respectively. ECEC characteristics included outdoor and indoor play environment quality, outdoor and indoor play equipment, screen-time environment quality, and policy environment quality. Mean (SD) scores for the PACER (*n* = 142) and CSMP (*n* = 91) were 3.7 ± 2.3 laps and 19.0 ± 5.5 criteria, respectively, which were moderately correlated with each other (Pearson r = 0.5; *p* < 0.001). Linear regression models revelated that a better policy environment score was associated with fewer PACER laps. Better outdoor play and screen-time environment quality scores and more outdoor play equipment were positively associated with higher CMSP scores. ECEC environments that reflect best practice guidelines may be opportunities for locomotor skills development in preschool-aged children. ClinicalTrials.gov Identifier: NCT03261492 (8/25/17).

## 1. Introduction

Locomotor skills development is an important part of early childhood growth that can enhance physical, cognitive, social, and emotional health [1]. In addition, it is hypothesized that there is a reciprocal relationship between motor development and physical activity, whereby participation in physical activity helps children to acquire and refine their locomotor skills, and locomotor skills acquisition reinforces continued participation in physical activity as children age [2,3]. Consequently, environments that promote locomotor skills development may have a substantial impact on the overall well-being of young children and reduce their risk for multiple major chronic diseases, i.e., obesity, cancer, diabetes, and cardiovascular disease, by providing them the skills they need to engage in developmentally appropriate and health-enhancing activities across the lifespan [4]. Further, locomotor development in childhood leads to more successful active play experiences and, ultimately, helps engage children in more activity opportunities during childhood, also reducing their risk of negative pediatric health outcomes (e.g., metabolic syndrome, anxiety, depression, asthma) [2,5].

Early care and education centers (ECECs) are one environment that affect children’s locomotor development. Preschool-age children spend over half of their waking hours at ECECs, and national position statements promote ECECs as an important setting for influencing health [6,7]. ECECs, however, are also the place where children are the most inactive throughout their typical week [8,9,10]. Further, activities that promote locomotor skills account for only one-fifth of young children’s school day activity opportunities [11,12]. Lack of opportunities to be active within ECECs have resulted in almost one-third of children having locomotor skills that fall short of developmental milestones by the time the children turn 3 years old [13]. Thus, ECEC environmental features that relate to positive locomotor outcomes need identification and replication in other ECECs to enhance activity opportunities in this critical setting, and ultimately, promote positive developmental and health outcomes.

Within the ECEC setting, larger classroom size/child ratio, lower population density of the surrounding area, a high level of teacher education, larger playground size, fewer field trips per month, and higher-quality screen time are all associated with better locomotor skills [14,15,16,17]. Further, higher-quality indoor and outdoor activity environments and enforced activity-related policies are associated with greater participation in physical activity at ECECs [10,18,19]. However, the correlation between physical activity and motor competence is low in early childhood, which suggests that environmental factors related to physical activity may have differential associations with locomotor outcomes at this age [2,13,20]. Thus, ECEC environmental characteristics need further study to examine their relationship with locomotor outcomes.

Locomotor outcomes are measured in two ways, product-based and process-based assessments, and no comprehensive, practical, and valid measures exist that can capture both types of assessments [21]. Product-based (quantitative) assessments evaluate the outcome of a movement, and process-based (qualitative) assessments evaluate how children perform a movement. In addition, quantitative outcomes (e.g., how many running laps can be completed) may not always be related to the quality of the movement (e.g., a child’s running form), and both types of assessments are needed to comprehensively assess the association between an environmental condition and locomotor outcomes [22]. To date, there are no established measures for assessing product-based locomotor outcomes in preschool-aged children; however, shuttle runs have face validity as an outcome measure and warrant further testing [11]. Details explaining the PACER test selection have been published elsewhere [23].

Thus, the purpose of this study was twofold. First, we examined the association between outdoor play, indoor play, screen-time, and policy ECEC environmental characteristics and locomotor outcomes, using product- and process-based assessments. Second, we compared the magnitude and direction of associations by type of locomotor assessment. We hypothesize that (1) several outdoor play, indoor play, screen-time, and policy environmental characteristics would be associated with locomotor outcomes, and (2) the magnitude of associations would differ by locomotor assessment type.

## 2. Materials and Methods

### 2.1. Participants

The analyses reported here are part of a supplementary project that added locomotor measures as part of the baseline assessments for the first two cohorts (2017–2018 and 2018–2019 academic years) of a three-cohort garden-based, obesity prevention intervention in 16 ECECs (28 ECECs in all three cohorts) throughout Phoenix, Arizona (ClinicalTrials.gov Identifier: NCT03261492). ECEC and participant recruitment methods for the intervention study have been published elsewhere [24]. Briefly, we recruited ECECs through previously established databases (e.g., Arizona licensed childcare facilities, Quality First program) and community partnerships. To be eligible, the ECEC must have (1) been licensed through the state of Arizona, (2) participated in the Child and Adult Care Food Program or National School Lunch Program, and (3) been located in a Hispanic/Latino-serving area (census tract with >30% Hispanic/Latino population). For each ECEC that agreed to participate, the director selected one classroom eligible for enrollment into the study. Directors chose classrooms based on the children’s age (3–5 years old) and the classroom teachers’ enthusiasm about receiving/utilizing a garden. All students in the classroom received the curriculum and all families were invited in the selected classroom to enroll in the study, but enrollment was limited to one parent and one child per family. Following an explanation of the study, parents provided informed consent for themselves and their child in English or Spanish. Children verbally assented to participate in motor skills protocols.

### 2.2. Instruments and Procedures

We used questions from the Behavioral Risk Factor Surveillance System survey to collect children’s date of birth, ethnicity, and sex [25]. We calculated age (months) as the difference between date of birth and the date of classroom assessments. Ethnicity was collapsed into Hispanic and non-Hispanic categories. Surveys were in either English or Spanish and in either an online or paper-based format.

#### 2.2.1. Height and Weight

As part of a baseline classroom visit, we obtained two measures of height (nearest 0.64 cm) and weight (nearest 0.22 kg) for each child, using a portable Seca Stadiometer (Model 213, Seca Corporation, Hamburg, Germany) and Tanita body composition analyzer (Model TBF-310, Tanita Corporation, Tokyo, Japan), respectively. Children removed their shoes and emptied their pockets before measurement. If measurements were more than 0.22 kg (i.e., 1/2 lb) or 0.64 cm (i.e.,1/4 in) apart, we collected a third measurement and averaged the two closest measurements. We calculated the body mass index (BMI) percentile, using the standard formula and CDC growth charts for children aged 2–5 years old [26].

#### 2.2.2. Environmental Assessments

We conducted a three-part environmental assessment at each ECEC to measure the quality of resources related to outdoor play, indoor play, screen-time, and policy environments, which included direct observation by research staff, self-report by teachers, and self-report by directors. A research team member assessed outdoor play environment quality (e.g., The outdoor play space for preschool children includes how many different areas for play?; The amount of outdoor play space that is shaded by structures or trees is: no shade, less than ¼ shaded, etc.), using seven questions from the outdoor play environment Nutrition and Physical Activity Self-Assessment for Child Care (NAP SACC) [27]. Questions were scored 0 (worst practice) to 3 (best practice) and summed to create an outdoor play environment quality score (range: 0–21).

One ECEC teacher per classroom reported on the outdoor play equipment total (i.e., Check all types of portable play equipment that are available and in good condition for children to use outdoors: swings, slides, etc.), indoor play equipment total, indoor play environment quality (i.e., The program offers the following in the indoor play space: gymnasium, separate play areas for each group, etc.), and screen-time environment quality (e.g., Televisions are located: In every classroom, In some classrooms, etc.). Teachers assessed indoor and outdoor play equipment, using a checklist that included 11 types of fixed play equipment and 11 types of portable play equipment combined from the NAP SACC and the Environment and Policy Assessment and Observation instruments [27]. Teachers reported the presence or absence of each item and whether the item was available inside, outside, or both. We summed the total number of items available outdoors (range: 0–22) and indoors (range: 0–22) to create play equipment scores.

Teachers also rated indoor play environment quality, using items from the NAP SACC. One item measured the availability and quality of space for physical activity (scored 0–3), and three items measured the availability of posters, books, and other learning materials that promote physical activity (each item scored 0–3). Teachers scored the availability of posters, books, and other learning materials that promote physical activity individually, but we averaged them based on the NAP SACC scoring protocol. We then added the mean score for the availability of posters, books, and other learning materials that promote physical activity to a score for the availability and quality of space for physical activity to derive a final indoor play environment quality score (range: 0–6). Teachers determined screen-time environment quality (range: 0–6) using two items from the screen time NAP SACC (scored 0–3).

An ECEC director reported the quality of outdoor play (7 components), screen time (6 components), and physical activity (8 components) policies by viewing three lists from NAP SACC and noting the presence or absence of each policy component. We calculated the total number of components present for each ECEC policy based on the NAP SACC scoring protocol. In addition, directors reported on the type of center that they managed, and we coded responses as Head Start or childcare center. Table 1 presents the best and worst practices for each item used to create the environmental assessment.

#### 2.2.3. Locomotor Skills

We used the Progressive Aerobic Cardiovascular Endurance Run (PACER) to measure children’s product-based locomotor skills [11]. The rationale for selection of the PACER test has been previously published [23]. We also used the locomotor portion of the Children’s Activity and Movement in Preschool Study (CHAMPS) motor skills protocol (CMSP) as a measure of process-based locomotor skills. We completed all testing at each ECEC in a single day during children’s recess period(s). Children completed the PACER test in groups of two or three after observing a demonstration. During the test, children ran back and forth 20 m with an initial running speed of 8.5 km/h, and a progressive 0.5 km/h increase in running speed every minute until they could not finish two laps in the required time or indicated that they were too tired to continue [28]. A research team member ran with the children to provide motivation, pacing, and instructions. Although the PACER is traditionally described as a measure of cardiovascular fitness, at the time of testing, it had been not validated as a measure of cardiovascular fitness in preschool-aged children; thus, we used it as a product-based measure of locomotor skills [29,30].

Due to the time required for administering the locomotor portion of the CMSP (about 5–10 min per child), only a subgroup of children completed it [31]. The classroom teacher selected the subgroup of children based on proximity, willingness to participate, and enrollment in the program. We tested as many children as possible during each one-day visit, and teachers determined the total number of children tested by the amount of time they made available for assessments. The locomotor portion of the CMSP consists of six skills (run, broad jump, slide, gallop, leap, and hop) that we scored twice based on three to seven criteria for each skill. Each child saw two expert demonstrations of the skill, one provided facing the child and one provided in the direction that the child was facing, before we asked the child to complete two trials of that skill. A trained assessor checked off each process-based characteristic (yes/no) that the child completed correctly. We averaged the two trials for each skill and summed the averages to obtain a total process-based locomotor score (range: 0–35). The CMSP is reliable (R = 0.88–0.97) and has strong concurrent validity with other locomotor assessments (R = 0.95–0.98) in preschool-aged children [31].

### 2.3. Data Analysis

We calculated a design effect (DEFF) and found low levels of clustering for both PACER laps (ICC = 0.04; DEFF = 1.44) and CMSP scores (ICC < 0.01; DEFF = 1.11), which prevented us from using multi-level modeling to assess the relationship between environmental variables and individual-level outcomes. However, when there is little variability at the ECEC level, other types of analyses (e.g., complex samples procedures, ecologic analysis) provide additional information about environmental factors that may be related to individual outcomes [32]. As we measured several independent variables at the ECEC level, we treated the data as arising from a complex sampling design (observations sampled from within ECECs) to adjust standard errors for clustering. ECEC was the clustering variable, sampling of observations at the within-ECEC level was assumed to be without replacement, and unit weighting was used [33].

We calculated descriptive statistics for the sample, including means, standard deviations, and percentages, and we used independent t-tests and chi-square tests to assess demographic differences by the test completed (CMSP vs. PACER only). For preliminary examination of the association between each environmental measure and locomotor outcomes, we estimated single-predictor general linear models (with adjustment for within-ECEC clustering). Next, we used linear regression models to determine important ECEC environmental characteristics related to each locomotor outcome (PACER or CMSP). We entered variables into models in up to three blocks: individual demographic variables (Model 1), ECEC center type (Model 2), and the six environmental characteristics (Model 3). We evaluated models after each block and removed non-significant variables at each stage based on *p* > 0.20. In the final model, we considered *p* < 0.05 as statistically significant. We completed all analyses using SPSS version 24.

## 3. Results

### 3.1. Study Sample

In this study, we obtained consent for 172 children (78 boys, mean ± standard deviation age 53.4 ± 4.4 months old; 75 girls, mean ± standard deviation age 52.9 ± 4.7 months old), and 142 and 91 children from 16 ECECs (range: 5–14 children per ECEC) completed the PACER test and CMSP test, respectively. Two children who completed the CMSP declined participation in the PACER test. One ECEC, comprised mainly of girls (87.5%), opted not to participate in the CMSP; thus, a higher proportion of boys (59.3%; *n* = 54) completed the CMSP than did girls (40.7%; *n* = 37; *p* = 0.01). No other demographic differences existed between those who completed the CMSP (*n* = 91) and those who did not (*n* = 53; *p* > 0.05). Children who completed the PACER (*n* = 142) test were (mean ± SD) 53.3 ± 4.5 months old, 49.3% female, 78.0% Hispanic, at a BMI percentile of 66.4 ± 29.4, and able to complete a mean of 3.7 ± 2.3 laps on the PACER test. Children who completed the CMSP test (*n* = 91) were 53.1 ± 4.4 months old, 40.7% female, 69.2% Hispanic, at a BMI percentile of 66.4 ± 28.5, and able to meet 19.0 ± 5.5 criteria on the CMSP test. PACER and CMSP scores were moderately correlated (Pearson r = 0.5; *p* < 0.001). ECECs (*n* = 16) were 62.5% Head Start-affiliated. The average ECEC environmental characteristic scores are presented in Table 1. Additional descriptive statistics of the full sample are presented by the child’s sex in Table 2.

### 3.2. Environmental Characteristics and Quantitative Locomotor Skills

In unadjusted bivariate models, indoor play equipment total was positively, but not significantly, associated with number of PACER laps (beta = 0.2; *p* = 0.06), and higher policy environment score was negatively associated with number of PACER laps (beta = −0.2; *p* = 0.03). The fully adjusted linear regression model revealed that children’s age and sex were associated with PACER laps in Models 1 and 3, and that policy environment quality was associated in Model 3 (Table 3). In Model 1, child sex and age explained 20.7% of variance in PACER laps; ECEC environmental characteristics (in Model 3) explained an additional 7.4%. In an effort to determine which policies relate to locomotor skills, we substituted individual policy scores (outdoor play, screen time, and physical activity) for ECEC policy quality in the final model, which revealed that physical activity policy quality was inversely associated with PACER laps (beta = −0.8; *p* = 0.01), but that screen time and outdoor play policies quality were not. Although the distribution of PACER scores approached normality, there was evidence of a small floor effect (i.e., a relatively high number of low or near-zero PACER scores). In sensitivity analysis, removal of the four most extreme PACER values (*n* = 4; 100% boys) restored normality but did not affect the results for the final models.

### 3.3. Environmental Characteristics and Quantitative Locomotor Skills

No ECEC environmental characteristics were associated with CMSP score in unadjusted bivariate models. A fully adjusted linear regression model revealed that children’s age and ethnicity were associated with CMSP in all models. Outdoor play environment quality, outdoor play equipment total, and screen-time environment quality were positively associated with CMSP in Model 3 (Table 4). In Model 1, child sex and ethnicity explained 17.6% of the variance in CMSP score; ECEC environmental characteristics (in Model 3) explained an additional 5.7%.

## 4. Discussion

The results of this study support a relationship between some characteristics of the ECEC environment and preschool-aged children’s locomotor skills. Related to our first hypothesis that several outdoor play, indoor play, screen-time, and policy environmental characteristics would be associated with locomotor outcomes, we found a negative association between physical activity policy quality and product-based motor skills. We also found that a higher-quality outdoor play environment, more outdoor play equipment, and higher-quality screen-time environments are associated with higher process-based locomotor skills. Indoor play environment quality and indoor play equipment totals were not associated with locomotor outcomes. Researchers, practitioners, and policymakers may want to compare and focus their efforts on studying and improving those environments that relate to multiple outcomes important for health (e.g., physical activity, sedentary behavior, motor skills), as changes to these environments have the potential to influence two important developmental outcomes. They may also be critically important in the prevention of pediatric chronic diseases and/or reduce the incidence of them; however, longitudinal studies are needed to examine these hypotheses further.

For the outdoor play environment, previous studies have shown that high population density of the surrounding area and larger playground size are associated with better locomotor skills [16,17]. We add to this work by showing that two facets of the outdoor environment, resources available (e.g., types of fixed and portable play equipment) and quality (e.g., shade, number of play areas, bike path quality), are associated with process-based locomotor outcomes. However, several outdoor play environment characteristics still had low scores in this study. Specifically, most ECECs did not have a garden, had limited shade, and had a small number of play areas. Low scores in these areas suggest that there are opportunities for improving the ECEC environment and, potentially, children’s locomotor development. Future studies could examine how improvements to these environmental features relate to improved locomotor skills development in preschool-aged children.

Also related to our first hypothesis, we observed that a better screen-time environment at the ECEC (i.e., fewer televisions and computers) was associated with higher locomotor skills. These findings conflict with True et al.’s study [17], which found that more electronic media usage was associated with higher locomotor skills in preschool-aged children. True et al., however, noted that socioeconomic status could have been a confounding factor in their analysis (e.g., higher-income children who attended ECECs with more access to electronic media). The current study includes center type as a proxy measure of socioeconomic status, but it was not a significant factor in the results. Further, screen-time policy quality was not a significant predictor of either locomotor outcome. More studies with stronger designs can help to determine the direction and strength of the association between the screen-time environment and locomotor outcomes.

Finally, contrary to our first hypothesis, we found a negative association between physical activity policy quality and product-based locomotor skills (i.e., PACER laps). We provide several potential explanations for this finding. First, previous research has found both positive and negative associations between ECEC policy quality and children’s participation in physical activity, and several of these studies cited implementation and enforcement of policies as potential reasons that policies were not translated into practice [18,34]. We did not measure policy implementation or enforcement in our study; thus, differential implementation and enforcement could have contributed to the negative associations between policy quality and locomotor skills that we found. Second, the criteria evaluated in the measure of physical activity policy quality (e.g., amount of time, types of clothes worn, not taking away physical activity as punishment) do not promote locomotor skill development but, instead, promote opportunities to be active. Although policies that create more opportunities for children to be active are important, more detailed policies that promote engagement in locomotor activities (e.g., running, jumping, skipping) may be needed to facilitate development of locomotor skills in preschool-aged children. Finally, we used the PACER test as a product-based measure of locomotor skills. Although PACER has face validity as a measure of product-based locomotor skills, other factors (e.g., children’s motivation and ability to understand directions, differences in test administration) also may contribute to a child’s PACER score. In this study, PACER test scores demonstrated a small floor effect (i.e., multiple scores of zero or one lap completed), suggesting that the measure was not sensitive enough to capture the full range of product-based outcomes. The presence of a floor effect limited outcome variability and may have resulted in a spurious association.

Related to our second hypothesis that the magnitude of associations will differ by locomotor assessment type, we found that none of the associations observed in this study were consistent for product- (i.e., PACER) and process-based (i.e., CSMP) outcome measures, despite moderate correlation between outcomes. We also found evidence of a floor effect for PACER test scores (i.e., a relatively high number of low or near-zero PACER scores), which suggests that it may not be a valid test for measuring locomotor skills in preschool-aged children. Despite suggestions that the shuttle run tests have face validity as a process-based outcome measure, we believe that it should not be used as a locomotor outcome measure with pre-school aged children, and researchers should use alterative measures, such as a dash test or average running speed, when assessing product-based locomotor skills [22,35]. Further, researchers, practitioners, and policymakers should weigh the added participant burden of using both product- and process-based outcome measures with the need for comprehensive locomotor assessment methods when deciding which outcome measures to include in their project.

One strength of this study is that it included a predominantly Hispanic sample, and we found that Hispanic preschool-aged children had lower CSMP scores than did their non-Hispanic peers. Although not the primary purpose of this analysis, these findings are consistent with other studies that assess locomotor skills across demographically different populations [15]. Further, Hispanic preschool-aged children often participate in less moderate-to-vigorous physical activity and have higher BMIs than do their non-Hispanic peers [36,37]. Consequently, our study highlights the need for future research to examine locomotor differences between Hispanic and non-Hispanic preschool-aged children, and to identify the relation between locomotor measures and developmental or health outcomes in Hispanic children. This study also used the NAP SACC self-assessments to examine the quality of the ECEC environment [27]. NAP SACC is a tool for practitioners to assess the quality of their own ECEC that is freely available (https://gonapsacc.org/self-assessment-materials; accessed on 4 February 2022) and easy to administer. As a result, ECEC teachers and directors can readily determine the quality of their ECEC’s environment, especially as they consider potential avenues to increase opportunities for locomotor skills.

As a limitation, teachers and directors self-reported several of the ECEC environmental factors prior to participation in an obesity prevention intervention. Use of self-report measures could have led to social desirability bias, in which environments were reported as being more conducive to locomotor skills development. Despite this limitation, the use of a crossover design allowed for all schools to receive the intervention; thus, we likely captured social desirability bias equally across all ECECs. The SAGE study also collected physical activity as an outcome measure [24], but because of its low correlation with locomotor outcomes in early childhood [13], we did not report those findings here. Future longitudinal studies should further explore and better understand the bi-directional relationship between physical activity and locomotor skills. Further, because this analysis was cross-sectional, we did not measure policy implementation, policy enforcement, or proximity of changes in the ECEC environment to the timing of the locomotor assessment. The timing of changes in the ECEC environment and level of policy implementation have the potential to influence the direction or magnitude of the association between the ECEC environment and locomotor outcomes. Future studies, using longitudinal designs, can help us to understand how timing affects these relationships more fully. Finally, this study used the PACER test as a product-based outcome for locomotor skills, which resulted in a floor effect. Following development of our study protocols (summer 2017), Mora-Gonzalez et al. [38] have proposed adaptations to remedy this limitation for very young children, including recommending that children run with two research staff (one in front and one behind), as opposed to one, and that the initial running speed be reduced from 8.5 km/h to 6.5 km/h. Based on our findings, we echo these recommendations.

## 5. Conclusions

Better outdoor play environment quality, better screen-time environment quality, and more outdoor play equipment were positively associated with higher CMSP scores. A better policy environment score was associated with fewer PACER laps. ECECs’ outdoor play and screen-time environments that reflect best practice guidelines may be opportunities for locomotor skills development in preschool-aged children, and subsequently, may be methods for improving the health of pediatric populations. Future studies with longitudinal designs, however, should examine these associations further.

## Figures and Tables

**Table 1:** NAP SACC assessment best practices, worst practices, and mean scores.

Environment/Quality	Best Practice	Worst Practice	Mean Score (*M ± SD*; *n* = 16)
Outdoor play environment (range: 0–21)			10.6 ± 3.7
Amount of shade	More than 3/4 shaded	No shade	1.4 ± 0.7
Size of open areas for outdoor, games, activities, and events	Large enough for all children to run around safely	Not available	2.4 ± 0.6
Number of play areas	8 or more play areas	1 or 2 play areas	1.3 ± 1.1
Availability of a garden	A garden that grows enough fruits or vegetables to provide children meals or snacks during one or more seasons	No garden	0.2 ± 0.5
Quality of wheeled-toy path	Paved and at least 5 feet wide	No path	1.9 ± 1.1
Shape of wheeled-toy path	Curved and looped	No path	1.7 ± 1.2
Type of wheeled-toy path connections ○ Connects to building entrances ○ Connects the building to play areas ○ Connects different play areas to each other	3 types of connections	No path	1.8 ± 1.2
Indoor play environment (range: 0–6)			3.8 ± 1.0
Indoor space features: ○ Space for all activities, including jumping, running, and rolling ○ Separate play areas for each age group ○ Areas that allow play for individuals, pairs, small groups, and large groups ○ Full access for children with special needs	3 or 4 features	None	2.8 ± 0.4
Collection of posters, books, and other learning materials that promote physical activity	A large variety with materials with items added or rotated seasonally.	Few or no materials	1.0 ± 0.7
Screen-time environment (range: 0–6)			2.9 ± 1.3
Televisions	No televisions, or televisions are stored outside of the classroom and not regularly available to the children	In every classroom	2.3 ± 1.2
Computers	No computers, or computers are stored outside of the classroom and not regularly available to the children	In every classroom	0.6 ± 1.1
Policy environment (range: 0–9)			3.8 ± 3.0
Outdoor play policy quality ○ Amount of outdoor playtime provided each day ○ Ensuring adequate total playtime on inclement weather days ○ Shoes and clothes that allow children and teachers to play outdoors in all seasons ○ Safe sun exposure for children, teachers, and staff ○ Not taking away outdoor playtime to manage challenging behaviors ○ Professional development on outdoor play and learning ○ Education for families on outdoor play and learning	6 or 7 topics	No topics or no policy	1.7 ± 1.1
Physical Activity Policy Quality ○ Amount of time provided each day for indoor and outdoor physical activity ○ Limiting long periods of seated time for children ○ Shoes and clothes that allow children and teachers to actively participate in physical activity ○ Teacher practices that encourage physical activity ○ Not taking away physical activity time or removing children from long periods of physically active playtime to manage challenging behaviors ○ Planned and informal physical activity education ○ Professional development on children’s physical activity ○ Education for families on children’s physical activity	7 or 8 topics	No topics or no policy	1.3 ± 1.1
Screen-time policy quality ○ Amount of screen time allowed ○ Types of programming allowed ○ Appropriate supervision and use of screen time in classrooms ○ Not using screen time as a reward or to manage challenging behaviors ○ Professional development on screen time Education for families on screen time	5 or 6 topics	No topics or no policy	0.8 ± 1.1

Note. Scores for availability of indoor and outdoor play equipment ranged from 0 to 22, based on the presence or absence of 22 items (e.g., ball play equipment, climbing structured, floor play equipment sandbox, see-saw), but are not displayed in the table.

**Table 2:** Comparison of descriptive characteristics by child sex.

Variable	Total Sample	Boys (*n* = 73)	Girls (*n* = 71)	*p*-Value
Child demographics				
Child’s age ^1^ (*M* ± *SD*)	53.2 ± 4.5	53.6 ± 4.2	52.8 ± 4.7	0.327
BMI percentile (*M* ± *SD*)	66.5 ± 29.2	66.5 ± 29.5	66.5 ± 29.2	0.997
Girls (%)	49.3			
Hispanic (%)	78.9	74.2	83.9	0.182
PACER Score (*M* ± *SD*)	3.7 ± 2.3	4.4 ± 2.5	3.0 ± 1.9	**<0.001**
CSMP Score (*M* ± *SD*)	19.0 ± 5.5	19.7 ± 5.6	18.1 ± 5.2	0.188

^1^ Months. Bolded *p*-values denote statistical significance.

**Table 3:** Stepwise linear regression for PACER laps.

Variable	Model 1		Model 3	
	Beta	*p*-Value	Beta	*p*-Value
Child demographics				
Child’s age ^1^	0.2	**<0.001**	0.2	**<0.001**
Female sex	−1.2	**0.017**	−1.2	**0.007**
ECEC environment				
Outdoor play equipment total			0.1	0.133
Policy environment			−0.2	**0.014**

^1^ Months. Betas are unstandardized. Bolded *p*-values denote statistical significance. Model 1—Individual demographic variables; Model 2—Model 1 + ECEC center type; Model 3—Model 2 + six environmental characteristics. No Model 2 variables were statistically significant.

**Table 4:** Stepwise linear regression for CMSP criteria.

Variable	Model 1		Model 3	
	Beta	*p*-Value	Beta	*p*-Value
Child demographics				
Child’s age ^1^	0.5	**0.004**	0.6	**<0.001**
Non-Hispanic child	1.9	**0.030**	2.4	**0.024**
ECEC environment				
Outdoor play environment			0.2	**0.033**
Outdoor play equipment			0.3	**<0.001**
Indoor play equipment			−0.3	0.102
Screen-time environment			0.6	**0.024**

^1^ Months. Betas are unstandardized. Bolded *p*-values denote statistical significance. Model 1—Individual demographic variables; Model 2—Model 1 + ECEC center type; Model 3—Model 2 + six environmental characteristics. No Model 2 variables were statistically significant.

## Data Availability

The datasets analyzed during the current study are available from the corresponding author on request.
